# Prediction of patients requiring intensive care for COVID-19: development and validation of an integer-based score using data from Centers for Disease Control and Prevention of South Korea

**DOI:** 10.1186/s40560-021-00527-x

**Published:** 2021-01-29

**Authors:** JoonNyung Heo, Deokjae Han, Hyung-Jun Kim, Daehyun Kim, Yeon-Kyeng Lee, Dosang Lim, Sung Ok Hong, Mi-Jin Park, Beomman Ha, Woong Seog

**Affiliations:** 1 The Armed Forces Medical Command, Ministry of National Defense, 81, Saemaeul-ro 177, Bundang-gu, Seongnam-si, Gyeonggi-do Republic of Korea; 2 Division of Pulmonary and Critical Care Medicine, Department of Internal Medicine, The Armed Forces Capital Hospital, Seongnam-si, Gyeonggi-do Republic of Korea; 3 Department of Periodontology, The Armed Forces Capital Hospital, Seongnam-si, Gyeonggi-do Republic of Korea; 4 Division of Chronic Disease Control, Korea Center for Disease Control and Prevention, Cheongju-si, Chungcheongbuk-do Republic of Korea

**Keywords:** COVID-19, Critical care, Prognosis

## Abstract

**Background:**

Unavailability or saturation of the intensive care unit may be associated with the fatality of COVID-19. Prioritizing the patients for hospitalization and intensive care may be critical for reducing the fatality of COVID-19. This study aimed to develop and validate a new integer-based scoring system for predicting patients with COVID-19 requiring intensive care, using only the predictors available upon triage.

**Methods:**

This is a retrospective study using cohort data from the Korean Centers for Disease Control and Prevention that included all admitted patients with COVID-19 between January 19 and June 3, 2020, in South Korea. The primary outcome was patients requiring intensive care defined as actual admission to the intensive care unit; at any time use of an extracorporeal life support device, mechanical ventilation, or vasopressors; and death. Patients admitted until March 20 were included for the training dataset to develop the prediction models and externally validated for the patients admitted afterward. Two logistic regression models were developed with different predictors and the predictive performance was compared: one with patient-provided variables and the other with added radiologic and laboratory variables. An integer-based scoring system was developed based on the developed logistic regression model.

**Results:**

A total of 5193 patients were considered, with 4663 patients included after excluding patients with age under 18 or insufficient data. For the training dataset, 3238 patients were included. Of the included patients, 444 (9.5%) patients required intensive care. The model developed with only the clinical variables showed an area under the curve of 0.884 for the validation set. The performance did not differ when radiologic and laboratory variables were added. Seven variables were selected for developing an integer-based scoring system: age, sex, initial body temperature, dyspnea, hemoptysis, history of chronic kidney disease, and activities of daily living. The area under the curve of the scoring system was 0.880.

**Conclusions:**

An integer-based scoring system was developed for predicting patients with COVID-19 requiring intensive care, with high performance. This system may aid decision support for prioritizing the patient for hospitalization and intensive care, particularly in a situation with limited medical resources.

**Supplementary Information:**

The online version contains supplementary material available at 10.1186/s40560-021-00527-x.

## Background

COVID-19 is a pandemic with over 12 million confirmed cases worldwide as of July 10, 2020. Death from the disease exceeded 500,000. Death rates differ among countries and even among cities, and medical resource availability may be a major factor of the differences [1]. The fast spread of the virus is causing excessive stress on the public health systems [2]. Efforts are being made to alleviate this stress by increasing medical supply [3], but it may not be enough for an overwhelming outbreak, especially for situations with limited medical resources.

COVID-19 is mild or asymptomatic in about 80–90% of cases [4]. Rates for the cases requiring intensive care are low, with 10–20% being admitted to the intensive care unit (ICU), 3–10% requiring intubation, and 2–5% dead [5]. Severe patients show respiratory failure, pneumonia, multi-organ failure, and shock, which require care in intensive care facilities. In those patients, care in ICU is critical for survival. While the mortality rate is variable among countries, unavailability or saturation of ICU is one of the crucial factors that affect the mortality [4]. ICU capacity differs among countries, lower in lower-middle-income countries [6]. Within the ICU, patients with COVID-19 should be admitted to an airborne infection isolation room to protect other admitted patients and medical staff from the transmission of COVID-19. As such, there are concerns about the limited availability of ICU facilities for patients with COVID-19.

While it is uncertain how many and which patients with COVID-19 need hospitalization and care in ICU, many patients with mild symptoms are often hospitalized due to a fear of aggravation and the necessity of quarantine. This may further exhaust medical resources including the availability of hospital beds under resource-limiting conditions. In this context, prioritizing patients with COVID-19 for care in a professional medical facility, especially for the ICU, may help reduce the mortality rate in COVID-19 epidemic hotspots.

The course of COVID-19 is variable. The median time from symptom onset to severe hypoxemia and ICU admission is approximately 7–12 days [6]. Although the rates of patients needing ICU care is currently unknown, 6.1% were classified as critical and 13.8% as severe [7]. However, it is often unpredictable who will need ICU care at the early stage of disease or presentation to the hospital [6]. Predicting the patients with COVID-19 at risk of death or needing ICU care may help prioritize the hospitalization of the patient at triage.

In this study, we aimed to develop and validate a model for predicting admission to ICU at presentation to the hospital using data from a nation-wide cohort of patients with COVID-19 in South Korea.

## Methods

### Study population

This was a retrospective study using cohort data that included all patients with COVID-19 in South Korea from 100 hospitals. The cohort was developed and managed by the Korean Centers for Disease Control and Prevention (KCDC). Patients with laboratory-confirmed COVID-19 were either admitted to a hospital or a community treatment center according to regional risk stratification and triage system [8]. Patients without any risk factors or severe symptoms were admitted to the community treatment center, and if any of those patients worsened afterwards, they were transferred to a hospital. KCDC mandated the hospitals to register their patients’ data to the cohort. Among the admitted patients, those who died or confirmed free of disease after management and released from quarantine from January 25, 2020, to June 3, 2020 were included in this study. Patients who were admitted until March 20, 2020, were used for the training dataset, and temporal external validation was performed on the patients admitted afterward. All patients were included for this study, except for those under 18 years old or those with incomplete data.

### Data and variables

Variables used for developing the outcome prediction model included those for the outcome, demographics, medical history, clinical symptoms and signs, imaging findings, and laboratory results. Admission to ICU; use of extracorporeal life support (ECLS), mechanical ventilation, or vasopressors; and death were included to derive the primary outcome. Demographic variables included age and sex. Medical history included pregnancy, diabetes, heart failure, hypertension, chronic cardiac disease, asthma, chronic obstructive pulmonary disease, chronic kidney disease, cancer, chronic liver disease, chronic neurologic disorders, chronic hematologic disorders, human immunodeficiency virus infection, dementia, smoking status, and activities of daily living (ADL) scale. Clinical variables included initial body temperature, cough, sputum, hemoptysis, sore throat, rhinorrhea, chest discomfort, myalgia, fatigue, and dyspnea. The only variable included from the imaging results was a binary value of whether there was any infiltration shown in the initial chest X-ray. Laboratory findings included counts of white blood cell, lymphocyte, and platelet and levels of hemoglobin, hematocrit, albumin, aspartate aminotransferase, alanine aminotransferase, blood urea nitrogen, and creatinine. Initial values that were acquired for the patients were used.

### Outcomes

The primary outcome was patients requiring intensive care defined as actual admission to the ICU; at any time use of an ECLS device, mechanical ventilation, or vasopressors; and death. Death was defined when the patient died during the follow-up period.

### Statistical analysis

Descriptive statistics were performed for all variables. Two logistic regression models were developed to assess the importance of input variables. These two models had the same prediction target as the patients requiring ICU care, but with different predictors. The first model was built only with variables that can be acquired during triage, which can easily be provided by the patient, including the patient’s demographics, medical history, clinical symptoms, and body temperature. The other model used radiologic findings and laboratory results in addition to all variables from the first model. The area under the curve (AUC) was calculated for the training set and the validation set to assess the predictive accuracy of the model.

From the two logistic regression models, variables that were statistically significant during the stepwise backward elimination were used to build the integer-based scoring model. Coefficients from the first model, which used only the variables that can be provided by the patient, were multiplied by 4 and rounded to the nearest integer to generate an integer-based scoring system. The overall score was calculated as the sum of the scores. The scoring system was applied to the validation dataset to acquire the accuracy of the model. All *P* values were 2-sided, and *P* < 0.05 was considered statistically significant. Statistical analysis was performed using R, and packages MASS and caret were used [9–11].

## Results

A total of 5193 patients with confirmed COVID-19 from 100 centers was registered to the cohort during the study period. Patients under 18 were excluded (117 patients), and those with insufficient data were excluded (413 patients), leaving 4663 patients for analysis. From the included patients, 444 (9.5%) patients required ICU care, 213 (4.6%) patients were actually admitted to ICU, and 217 (4.7%) patients died. A total of 3238 patients were admitted before March 20, 2020, and used to develop the prediction models. Patients admitted after were used to validate the developed model, and 1425 patients were included (Table 1).

**Table 1:** Comparison between the training set and the validation set

	Total (***n*** = 4663)	Training set (***n*** = 3238)	Validation set (***n*** = 1425)	***P*** value
Demographic				
Age	55.0 [37.0;67.0]	56.0 [41.0;68.0]	53.0 [30.0;66.0]	< 0.001
Sex, male	1841 (39.5%)	1182 (36.5%)	659 (46.2%)	< 0.001
Symptoms and signs				
Initial body temperature	36.8 [36.5;37.3]	36.9 [36.5;37.3]	36.8 [36.5;37.2]	0.001
Cough	1944 (41.7%)	1523 (47.0%)	421 (29.5%)	< 0.001
Sputum	1344 (28.8%)	1049 (32.4%)	295 (20.7%)	< 0.001
Hemoptysis	26 (0.6%)	23 (0.7%)	3 (0.2%)	0.058
Sore throat	682 (14.6%)	514 (15.9%)	168 (11.8%)	< 0.001
Rhinorrhea	440 (9.4%)	334 (10.3%)	106 (7.4%)	0.002
Chest pain	351 (7.5%)	294 (9.1%)	57 (4.0%)	< 0.001
Myalgia	733 (15.7%)	583 (18.0%)	150 (10.5%)	< 0.001
Arthralgia	18 (0.4%)	16 (0.5%)	2 (0.1%)	0.124
Lethargic	179 (3.8%)	143 (4.4%)	36 (2.5%)	0.003
Dyspnea	632 (13.6%)	528 (16.3%)	104 (7.3%)	< 0.001
Headache	773 (16.6%)	612 (18.9%)	161 (11.3%)	< 0.001
Nausea, vomiting	216 (4.6%)	171 (5.3%)	45 (3.2%)	0.002
Diarrhea	400 (8.6%)	333 (10.3%)	67 (4.7%)	< 0.001
Medical history				
Pregnancy	17 (0.4%)	12 (0.4%)	5 (0.4%)	1.000
Diabetes	704 (15.1%)	510 (15.8%)	194 (13.6%)	0.067
Heart failure	66 (1.4%)	40 (1.2%)	26 (1.8%)	0.151
Hypertension	1183 (25.4%)	847 (26.2%)	336 (23.6%)	0.068
Chronic cardiac disease	188 (4.0%)	138 (4.3%)	50 (3.5%)	0.261
Asthma	118 (2.5%)	93 (2.9%)	25 (1.8%)	0.033
COPD	40 (0.9%)	34 (1.1%)	6 (0.4%)	0.048
Chronic kidney disease	56 (1.2%)	45 (1.4%)	11 (0.8%)	0.101
Cancer	154 (3.3%)	111 (3.4%)	43 (3.0%)	0.526
Chronic liver disease	72 (1.5%)	51 (1.6%)	21 (1.5%)	0.897
Chronic neurologic disorder	41 (0.9%)	23 (0.7%)	18 (1.3%)	0.091
Chronic hematologic disorder	34 (0.7%)	27 (0.8%)	7 (0.5%)	0.280
HIV infection	9 (0.2%)	7 (0.2%)	2 (0.1%)	0.856
Autoimmune disease	32 (0.7%)	25 (0.8%)	7 (0.5%)	0.380
Dementia	320 (6.9%)	172 (5.3%)	148 (10.4%)	< 0.001
Smoking				< 0.001
Never smoker	4280 (91.8%)	3034 (93.7%)	1246 (87.4%)	
Ex-smoker	131 (2.8%)	94 (2.9%)	37 (2.6%)	
Current smoker	252 (5.4%)	110 (3.4%)	142 (10.0%)	
ADL				< 0.001
Normal	4011 (86.0%)	2884 (89.1%)	1127 (79.1%)	
Partially dependent	358 (7.7%)	194 (6.0%)	164 (11.5%)	
Totally dependent	294 (6.3%)	160 (4.9%)	134 (9.4%)	
Imaging and laboratory findings				
Chest X-ray infiltration	1650 (35.4%)	1229 (38.0%)	421 (29.5%)	< 0.001
Hemoglobin level, g/dL	13.2 [12.1;14.4]	13.2 [12.2;14.3]	13.3 [12.1;14.5]	0.066
Platelet count, 10^3^/μL	224.0 [176.0;280.0]	227.0 [178.0;283.0]	218.0 [175.0;273.0]	0.002
WBC count, 10^3^/μL	5.7 [4.4;7.1]	5.7 [4.5; 7.2]	5.7 [4.4; 7.0]	0.301
Lymphocyte, %	28.7 [20.9;36.2]	28.7 [20.9;36.1]	28.6 [21.3;36.5]	0.638
Hematocrit, %	39.1 [36.2;42.3]	39.0 [36.1;42.1]	39.5 [36.2;42.8]	0.018
AST level, IU/L	24.0 [19.0;33.0]	25.0 [20.0;34.0]	23.0 [19.0;32.0]	< 0.001
ALT level, IU/L	20.0 [14.0;31.0]	20.0 [14.0;31.0]	19.0 [13.0;30.0]	< 0.001
Albumin level, g/dL	4.2 [3.8;4.5]	4.1 [3.8; 4.4]	4.3 [3.9; 4.6]	< 0.001
BUN level, mg/dL	12.3 [10.0;15.8]	12.5 [10.0;16.0]	12.0 [9.7;15.0]	< 0.001
Creatinine level, mg/dL	0.7 [0.6;0.9]	0.7 [0.6; 0.9]	0.8 [0.6; 0.9]	0.342
Outcomes				
Requiring ICU	444 (9.5%)	320 (9.9%)	124 (8.7%)	0.226
Admission to ICU	213 (4.6%)	113 (3.5%)	70 (5.0%)	0.016
Mechanical ventilation	49 (1.1%)	35 (1.1%)	14 (1.0%)	0.697
ECLS	28 (0.6%)	22 (0.7%)	6 (0.4%)	0.397
Vasopressor treatment	118 (2.5%)	83 (2.6%)	35 (2.5%)	0.182
Death	217 (4.7%)	141 (4.3%)	76 (5.4%)	0.113

Values are number (%) or median [interquartile range]

*ADL* activities of daily living, *AST* aspartate aminotransferase, *ALT* alanine aminotransferase, *BUN* blood urea nitrogen, *COPD* chronic obstructive pulmonary disease, *ECLS* extracorporeal life support, *HIV* human immunodeficiency virus, *ICU* intensive care unit, *WBC* white blood cell

### Accuracy of the two prediction models

**Fig. 1:**
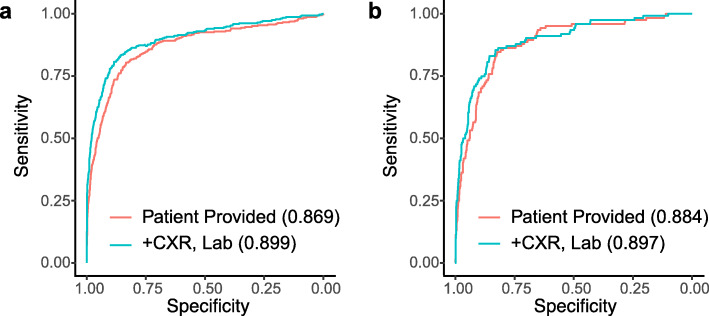
Receiver operating characteristic curves for **a** the training and **b** the validation dataset. The area under the curves shown in parenthesis

Multivariate logistic regression models were built with stepwise backward elimination of insignificant variables. The first model, developed with only the variables that a patient can provide showed an AUC of 0.869 [95% CI 0.845–0.893] for the training set and 0.884 [95% CI 0.852–0.916] for the validation set. The second model, which included the findings from the initial chest X-ray and the laboratory results in addition to the first model, showed a slightly increased accuracy (AUC 0.899 [95% CI 0.877–0.920] for the training set and AUC 0.897 [95% CI 0.865–0.928] for the validation set) but was not significant (*P* = 0.169) (Fig. 1).

### Integer-based scoring system

Using the multivariate logistic regression analysis results from the first model, a total of seven variables were selected as significant predictors for the patients requiring ICU care: sex, age, initial body temperature on admission, dyspnea, hemoptysis, history of chronic kidney disease, and ADL scale (Table 2). The mean variable inflation factor was 1.08 (range 1.01–1.24), showing low collinearity. The coefficients of the model were multiplied by 4 and rounded to the nearest integer, to form an integer-based scoring system to predict patients with COVID-19 requiring ICU care (COVIC score). Continuous variables (age and initial body temperature) were categorized, with the median value used to calculate the score point for the category. For the continuous variables, the score for each category was subtracted by the score of the lowest category, for ease of calculation. As a result, the score of the lowest category was set to zero for age and temperature (Table 3).

**Table 2:** Predictors of patients requiring intensive care unit care in multivariate logistic regression

Variables	Odds ratio	Lower 95% CI	Upper 95% CI	Std error
Age^a^	1.039	1.029	1.050	0.005
Sex, female	0.369	0.280	0.487	0.141
Initial body temperature on admission^a^	1.506	1.234	1.835	0.101
Hemoptysis	5.371	1.737	15.539	0.554
Dyspnea	5.124	3.846	6.834	0.147
Chronic kidney disease	4.119	1.921	8.714	0.385
ADL score 1^b^	4.113	2.751	6.122	0.204
ADL score 2^b^	8.753	5.771	13.285	0.212

*CI* confidence interval, *Std Error* standard error, *ADL* activities of daily living

^a^Continuous variables

^b^ADL score of 1 represents partially dependent and 2 represents totally dependent on others

**Table 3:** Scoring system to predict COVID-19 patients requiring ICU care (COVIC score)

Variables		Score
Age, years	18 to less than 30	0
	30 to less than 40	1
	40 to less than 50	3
	50 to less than 60	4
	60 to less than 70	6
	≥ 70	7
Male sex		4
Initial body temperature on admission, °C	< 37.0	0
	37.0 to less than 37.5	1
	37.5 to less than 38.0	2
	38.0 to less than 39	3
	≥ 39.0	4
Hemoptysis		7
Dyspnea		7
Chronic kidney disease		6
Activities of daily living^a^	1—partially dependent	6
	2—totally dependent	9

^a^Activities of daily living score of 1 represents partially dependent and 2 represents totally dependent on others

**Fig. 2:**
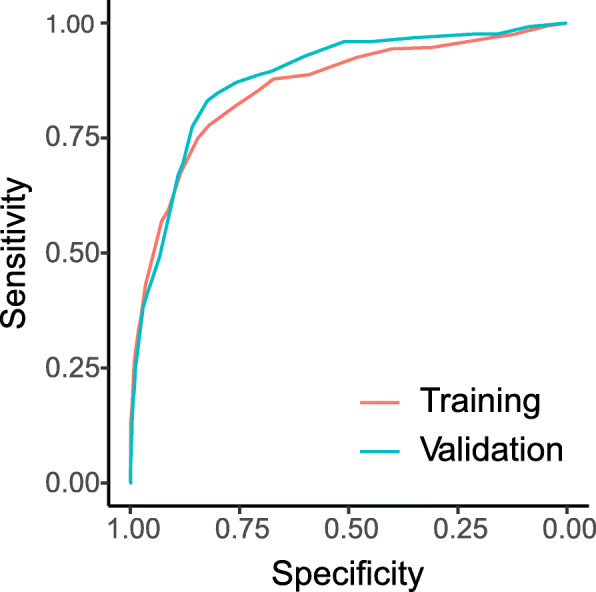
Receiver operating characteristic curve of the newly developed scoring system (COVIC score). Score tested on the validation dataset for predicting patients with COVID-19 requiring ICU care (COVIC score). The area under the curve was 0.880 [95% CI, 0.847–0.912]

**Fig. 3:**
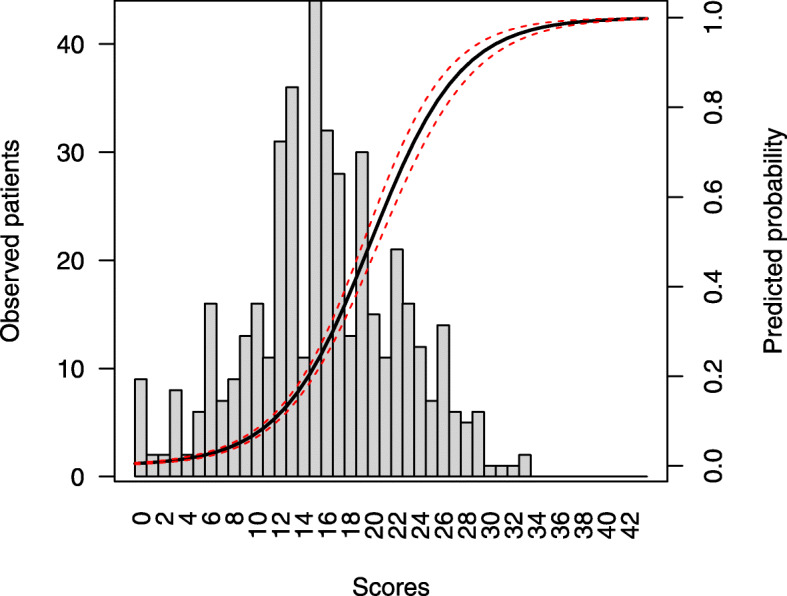
Association between the integer-based score and the probability of the patient requiring intensive care. The red dotted line indicates the 95% confidence interval

**Fig. 4:**
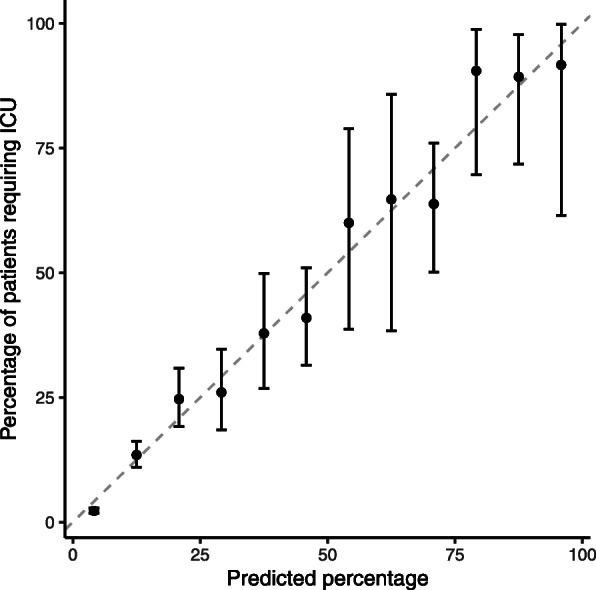
Calibration plot for the integer-based prediction score (COVIC score)

The performance of the newly developed scoring system was externally validated. The receiver operating characteristic curve was drawn with an AUC of 0.880 [95% CI, 0.847–0.912] (Fig. 2). The association between the integer-based score and the probability of the patient requiring ICU care for the total dataset is presented in Fig. 3. The Hosmer-Lemeshow test was performed with the chi-squared value of 8.866 and the *P* value of 0.35, suggesting goodness of fit (Fig. 4).

For better accessibility and ease of use, a web-based application was developed (see Additional file 2). The application can be accessed at http://covic.docl.org.

## Discussion

This study presents and validates an integer-based scoring system for prediction of patients with COVID-19 requiring ICU care (COVIC score), with excellent prediction performance. The scoring system consists of seven variables: sex, age, initial body temperature, hemoptysis, dyspnea, history of chronic kidney disease, and ADL scale.

Previous studies have shown that male sex, old age, and comorbidities including active cancer, coronary artery disease, liver and kidney dysfunctions, chronic obstructive pulmonary disease, diabetes, and hypercholesterolemia were factors associated with mortality among patients with COVID-19 who were admitted to ICU [12, 13]. These findings and ours suggest that patients with COVID-19 may have an increased risk of grave outcome or need intensive care when they are old and male and have comorbidities. Our findings suggest that additional presence of some symptoms and signs such as fever, hemoptysis, dyspnea, and dependent activities in daily living should be considered as a clue that patients may need intensive care during the course.

There were previous studies to predict patient outcomes for COVID-19 [14–18]. Mortality risk, hospital stay, and progression to severe state were the primary outcome for the prediction models [14–25]. One of the largest cases included were 1590 cases from a national retrospective cohort from China [18]. In that study, the scoring system included 10 variables, which were chest radiographic abnormality, age, hemoptysis, dyspnea, unconsciousness, number of comorbidities, cancer history, and laboratory values. Some of them are similar to variables in our scoring system but included those that can only be assessed after professional care, such as imaging results and laboratory values.

In this study, logistic regression analysis showed that additional variables including chest X-ray findings and laboratory results may not significantly increase the performance of the prediction for patients requiring ICU care. The variables that constitute the developed scoring system from this study do not require professional medical assessment but can be provided by the patient. As such, this scoring system may help to prioritize the patient for hospitalization at triage.

The scoring system in this study can be applied to wider usage. For instance, when the diagnostic capacity or hospital beds are insufficient to meet the demand of the patients with COVID-19, prioritizing the patient for hospitalization may be critical. If the patient is hospitalized on a first-come-first-served basis, then patients who may require ICU care during the course, but who is slower in arrival to the clinics, may not be able to receive proper management. In addition, this scoring system may help the paramedics transfer the patients suspected of COVID-19 to the appropriate hospital. Even for the undiagnosed patients, if needed, the feature that can be self-calculated by the patient may be useful.

In this study, the primary outcome, patients requiring ICU care, was defined as actual admission to the ICU; at any time use of an ECLS device, mechanical ventilation, or vasopressors; and death. Rather than choosing the patients admitted to the ICU, the sum of the patients listed above was chosen as the primary outcome since there were many hospitals with negative pressure quarantine rooms that substituted the ICU in case of saturated or lacked ICU in South Korea. In addition, the use of an ECLS device, mechanical ventilation, or vasopressors is an ample indicator of needing intensive care.

There are many factors that should be considered for the decision of needing ICU care in a patient. Only half (46%) of the patients actually admitted to the ICU died or used either an ECLS device, mechanical ventilation, or vasopressors. Descriptive analysis was performed for the patients admitted to the ICU (see Additional file 1). Oxygen supply was required for the 77 (66.9%) patients, with a significantly higher percentage of lymphocyte (17.9% vs. 12.1%, *P* value < 0.001) for the patients who did not use an ECLS device, mechanical ventilation, or vasopressors. However, the analysis results show that there are patients who were admitted to the ICU for causes that were not evident from our dataset.

The dataset used for this study was provided by the KCDC, with all admitted cases in South Korea subject to registration for the cohort. The remaining patients with COVID-19 were managed in a community treatment center. As a result, 56% (5193 out of 9306) of all South Korean patients with COVID-19 during the study period were analyzed for this study. Fortunately, the hospital beds are relatively sufficient in South Korea, and the maximum number of confirmed cases per day being 1062 cases (March 1, 2020). This ensured that the patients residing outside of the hospitals were neither critical nor needed hospitalization. For instance, anyone older than 65 years or with a chronic comorbidity such as diabetes were mandated to be admitted to a hospital by KCDC guidelines [26]. No confirmed patients were residing at home during the study period. As a result, there should be a very low risk of selection bias of the cohort that this study is based on.

This study has several limitations. While there are discrepancies in medical infrastructure between countries, this study was conducted in one country with ethnic homogeneity (98.2% were of Korean ethnicity). In addition, this was a retrospective study. Therefore, external validation for a different nation or ethnicity will be needed. About 8% of patients were excluded from developing the models due to insufficient data or young age. All symptom data were acquired upon admission and consideration for the onset of symptoms could have been more informative. The decision of ICU admission can be affected by the availability of ICU beds. Studying the association between the decision of ICU admission and the regional patient count would have been beneficial. However, this study could not consider the availability of ICU beds as a factor due to the limitation of the dataset. There could have been patients that were not accounted for that should have been admitted to the ICU but did not due to restrictive availability of ICU beds. The extended definition of primary outcome that included patients who died or needed an ECLS device, mechanical ventilation, or vasopressors were used to consider these patients. However, considering that only half of the patients admitted to the ICU met the criteria, there may be more.

## Conclusions

An integer-based scoring system was developed for the prediction of patients with COVID-19 requiring ICU care, with high performance. The developed model may be able to aid decision support for prioritizing the patient for hospitalization, especially for the circumstances where medical resources are limited.

## Supplementary Information

**Additional file 1: Additional Table I.** Descriptive statistics of the patients admitted to the intensive care unit. Description of data: A table presenting descriptive statistics of the patients admitted to the intensive care unit. A comparison between the patients who died or required an ECLS device, mechanical ventilator, or vasopressors and the patients who did not is shown.

**Additional file 2: Additional Figure I.** Screenshots of the web application developed for ease of use of the COVIC score. Description of data: Screenshots of the web application that calculates the result from the machine learning model presented in this study. Accessible at http://covid.docl.org.

## Data Availability

The datasets generated and/or analyzed during the current study are available in the Korean Centers for Disease Control and Prevention’s COVID-19 Clinical Epidemiologic Information Repository, https://www.cdc.go.kr/board/board.es?mid=a20504000000&bid=0014.

## Abbreviations

ADL: Activities of daily living
AUC: Area under the curve
ECLS: Extracorporeal life support
ICU: Intensive care unit
KCDC: Korean Centers for Disease Control and Prevention
