# Supplementary material for: Prediction of patients requiring intensive care for COVID-19: development and validation of an integer-based score using data from Centers for Disease Control and Prevention of South Korea
Source: J Intensive Care. 2021 Jan 29;9:16. doi: 10.1186/s40560-021-00527-x (PMC7844778; doi:10.1186/s40560-021-00527-x)
Supplement: Supplementary file 1 — Additional file 1: Additional Table I. Descriptive statistics of the patients admitted to the intensive care unit. Description of data: A table presenting descriptive statistics of the patients admitted to the intensive care unit. A comparison between the patients who died or required an ECLS device, mechanical ventilator, or vasopressors and the patients who did not is shown. [file 40560_2021_527_MOESM1_ESM.docx]

**Prediction of patients requiring intensive care for COVID-19: development and validation of an integer-based score using data from South Korea.**

ADDITIONAL FILE 1

Additional Table I. Descriptive statistics of the patients admitted to the intensive care unit.

Additional Table I. Descriptive statistics of the patients admitted to the intensive care unit.

|  | **Total (n=213)** | **Did not use an ECLS/mechanical ventilator/vasopressor or died (n=115)** | **Used an ECLS/ mechanical /vasopressor or died (n=98)** | ***P* Value** |
| --- | --- | --- | --- | --- |
| Demographic | | | | |
| Age | 71.0 [61.0;79.0] | 66.0 [57.0;78.0] | 73.0 [66.0;80.0] | <0.001 |
| Sex, Male | 122 (57.3%) | 61 (53.0%) | 61 (62.2%) | 0.225 |
| Symptoms and signs | | | | |
| Initial body temperature | 37.1 [36.5;37.8] | 37.1 [36.5;37.5] | 37.0 [36.5;37.9] | 0.373 |
| Cough | 98 (46.0%) | 59 (51.3%) | 39 (39.8%) | 0.123 |
| Sputum | 74 (34.7%) | 42 (36.5%) | 32 (32.7%) | 0.655 |
| Hemoptysis | 5 (2.3%) | 2 (1.7%) | 3 (3.1%) | 0.856 |
| Sore throat | 19 (8.9%) | 13 (11.3%) | 6 (6.1%) | 0.280 |
| Rhinorrhea | 14 (6.6%) | 7 (6.1%) | 7 (7.1%) | 0.974 |
| Chest pain | 12 (5.6%) | 8 (7.0%) | 4 (4.1%) | 0.543 |
| Myalgia | 33 (15.5%) | 21 (18.3%) | 12 (12.2%) | 0.308 |
| Arthralgia | 2 (0.9%) | 1 (0.9%) | 1 (1.0%) | 1.000 |
| Lethargic | 17 (8.0%) | 8 (7.0%) | 9 (9.2%) | 0.731 |
| Dyspnea | 98 (46.0%) | 45 (39.1%) | 53 (54.1%) | 0.041 |
| Headache | 23 (10.8%) | 16 (13.9%) | 7 (7.1%) | 0.172 |
| Nausea, vomiting | 12 (5.6%) | 8 (7.0%) | 4 (4.1%) | 0.543 |
| Diarrhea | 21 (9.9%) | 12 (10.4%) | 9 (9.2%) | 0.940 |
| Medical history | | | | |
| Pregnancy | 0 (0%) | 0 (0%) | 0 (0%) |  |
| Diabetes | 72 (33.8%) | 23 (20.0%) | 49 (50.0%) | <0.001 |
| Heart failure | 9 (4.2%) | 2 (1.7%) | 7 (7.1%) | 0.107 |
| Hypertension | 107 (50.2%) | 52 (45.2%) | 55 (56.1%) | 0.147 |
| Chronic cardiac disease | 19 (8.9%) | 10 (8.7%) | 9 (9.2%) | 1.000 |
| Asthma | 7 (3.3%) | 3 (2.6%) | 4 (4.1%) | 0.829 |
| COPD | 2 (0.9%) | 1 (0.9%) | 1 (1.0%) | 1.000 |
| Chronic kidney disease | 12 (5.6%) | 3 (2.6%) | 9 (9.2%) | 0.076 |
| Cancer | 18 (8.5%) | 7 (6.1%) | 11 (11.2%) | 0.273 |
| Chronic liver disease | 3 (1.4%) | 1 (0.9%) | 2 (2.0%) | 0.889 |
| Chronic neurologic disorder | 5 (2.3%) | 0 (0.0%) | 5 (5.1%) | 0.046 |
| Chronic hematologic disorder | 3 (1.4%) | 2 (1.7%) | 1 (1.0%) | 1.000 |
| HIV infection | 2 (0.9%) | 2 (1.7%) | 0 (0.0%) | 0.549 |
| Autoimmune disease | 2 (0.9%) | 0 (0.0%) | 2 (2.0%) | 0.409 |
| Dementia | 29 (13.6%) | 15 (13.0%) | 14 (14.3%) | 0.950 |
| Smoking |  |  |  | 0.691 |
| (Never smoker) | 186 (87.3%) | 101 (87.8%) | 85 (86.7%) |  |
| (Ex-smoker) | 21 (9.9%) | 10 (8.7%) | 11 (11.2%) |  |
| (Current smoker) | 6 (2.8%) | 4 (3.5%) | 2 (2.0%) |  |
| ADL |  |  |  | <0.001 |
| (Normal) | 121 (56.8%) | 80 (69.6%) | 41 (41.8%) |  |
| (Partially dependent) | 52 (24.4%) | 21 (18.3%) | 31 (31.6%) |  |
| (Totally dependent) | 40 (18.8%) | 14 (12.2%) | 26 (26.5%) |  |
| Imaging and laboratory findings |  |  |  |  |
| Chest X-ray infiltration | 147 (69.0%) | 71 (61.7%) | 76 (77.6%) | 0.019 |
| Hemoglobin level, g/dL | 12.4 ± 2.2 | 12.7 ± 2.1 | 12.0 ± 2.3 | 0.017 |
| Platelet count, 10^3^/µL | 183.0 [144.0;237.0] | 192.0 [149.5;230.5] | 170.0 [129.0;261.0] | 0.287 |
| WBC count, 10^3^/µL | 6.2 [4.5;8.1] | 5.7 [ 4.2; 7.1] | 7.0 [ 5.0; 9.6] | 0.001 |
| Lymphocyte, % | 15.0 [9.1;25.2] | 17.9 [11.2;26.5] | 12.1 [ 7.0;20.6] | 0.001 |
| Hematocrit, % | 36.5 ± 6.3 | 37.4 ± 6.0 | 35.4 ± 6.5 | 0.019 |
| AST level, IU/L | 38.0 [25.0;58.0] | 31.0 [24.0;47.5] | 48.0 [28.0;69.0] | 0.001 |
| ALT level, IU/L | 24.0 [14.0;38.0] | 22.0 [15.5;35.0] | 26.0 [14.0;39.0] | 0.267 |
| Albumin level, g/dL | 3.4 [3.0;3.8] | 3.5 [ 3.2; 4.1] | 3.3 [ 2.9; 3.6] | <0.001 |
| BUN level, mg/dL | 17.5 [12.0;24.7] | 14.6 [10.1;20.3] | 20.6 [14.0;30.0] | <0.001 |
| Creatinine level, mg/dL | 0.9 [0.7;1.1] | 0.8 [ 0.6; 1.0] | 1.0 [ 0.7; 1.4] | <0.001 |
| Outcomes |  |  |  |  |
| Oxygen supply requirement |  |  |  | <0.001 |
| Oxygen supply with nasal prong | 66 (31.0%) | 49 (42.6%) | 17 (17.3%) |  |
| Oxygen supply with facial mask | 52 (24.4%) | 22 (19.1%) | 30 (30.6%) |  |
| Non-invasive ventilation | 32 (15.0%) | 6 (5.2%) | 26 (26.5%) |  |
| Mechanical ventilation | 23 (10.8%) | 0 (0.0%) | 23 (23.4%) |  |
| ECLS | 2 (0.9%) | 0 (0.0%) | 2 (2.0%) | 0.285 |
| Vasopressor treatment | 35 (16.4%) | 0 (0.0%) | 35 (35.7%) | <0.001 |
| Death | 73 (34.3%) | 0 (0.0%) | 73 (74.5%) | <0.001 |

*ADL* activities of daily living, *AST* aspartate aminotransferase, *ALT* alanine aminotransferase, *BUN* blood urea nitrogen, *COPD* chronic obstructive pulmonary disease, *ECLS* extracorporeal life support, *HIV* human immunodeficiency virus, *ICU* intensive care unit, *WBC* white blood cell.

Values are number (%), median [interquartile range], or mean ± standard deviation.
