# Supplementary material for: Prediction of patients requiring intensive care for COVID-19: development and validation of an integer-based score using data from Centers for Disease Control and Prevention of South Korea
Source: J Intensive Care. 2021 Jan 29;9:16. doi: 10.1186/s40560-021-00527-x (PMC7844778; doi:10.1186/s40560-021-00527-x)
Supplement: Supplementary file 2 — Additional file 2: Additional Figure I. Screenshots of the web application developed for ease of use of the COVIC score. Description of data: Screenshots of the web application that calculates the result from the machine learning model presented in this study. Accessible at http://covid.docl.org. [file 40560_2021_527_MOESM2_ESM.docx]

**Prediction of patients requiring intensive care for COVID-19: development and validation of an integer-based score using data from South Korea.**

ADDITIONAL FILE 2

Additional Figure I. Screenshots of the web application developed for ease of use of the COVIC score.

**Additional Figure I.** **Screenshots of the web application developed for ease of use of the COVIC score.**

**
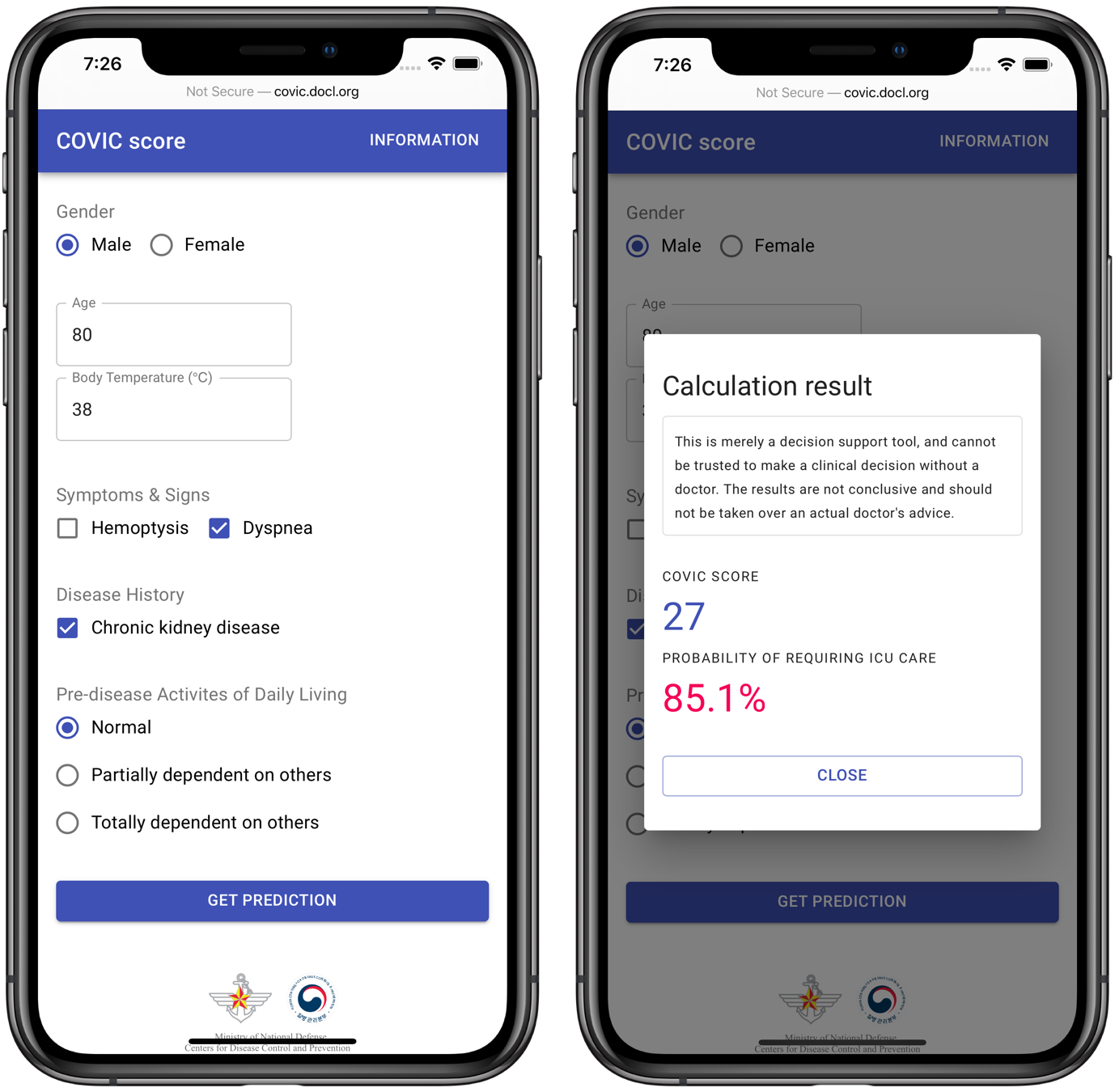
**

Accessible on the web on a mobile device or a desktop at [http://covic.docl.org](http://covid.docl.org/)
